# Lithium in Bipolar Disorder: Renal Mechanisms, Nephrotoxicity Phenotypes, and a Shared-Care Pathway for Clinical Practice

**DOI:** 10.3390/ijms27156730

**Published:** 2026-07-28

**Authors:** Nikolina Rijavec, Željka Večerić-Haler

**Affiliations:** 1 University Psychiatric Clinic Ljubljana, Chengdujska cesta 45, 1260 Ljubljana, Slovenia; nikolina.rijavec@psih-klinika.si; 2 Department of Psychiatry, Faculty of Medicine, University of Ljubljana, Vrazov trg 2, 1000 Ljubljana, Slovenia; 3 Department of Nephrology, University Medical Centre Ljubljana, Zaloška 2, 1000 Ljubljana, Slovenia; 4 Department of Internal Medicine, Faculty of Medicine, University of Ljubljana, Vrazov trg 2, 1000 Ljubljana, Slovenia

**Keywords:** lithium, bipolar disorder, chronic kidney disease, diabetes insipidus, nephrogenic, aquaporin 2

## Abstract

Lithium remains a cornerstone mood stabilizer for bipolar disorder, with strong evidence for relapse prevention and a unique association with reduced suicide risk. Its benefit is counterbalanced by renal adverse effects, ranging from impaired urinary concentrating capacity and nephrogenic diabetes insipidus (NDI) to chronic tubulointerstitial nephropathy with progressive loss of glomerular filtration rate in a minority of long-term users. Mechanistically, lithium enters collecting duct principal cells via the epithelial sodium channel, disrupts vasopressin-regulated water handling by downregulating aquaporin-2, and can drive chronic interstitial injury. Risk is amplified by episodes of lithium intoxication, dehydration, interacting medications that reduce renal lithium clearance, longer treatment duration, and comorbid kidney disease. This review integrates psychiatric and nephrologic perspectives on lithium’s indications, mechanisms, renal phenotypes, and prevention strategies, and proposes a practical shared-care pathway for patients with bipolar disorder who develop polyuria, NDI, acute kidney injury, or chronic kidney disease during lithium therapy, emphasizing monitoring, targeted treatment of polyuria, mitigation of toxicity, and structured shared decision-making when renal function declines.

## 1. Introduction: Why Lithium Still Matters (And Why Kidneys Matter)

Lithium remains among the most effective maintenance treatments for bipolar disorder and is consistently recommended as a first-line option across phases of illness in major guidelines [1]. Its clinical value includes relapse prevention and a reproducible association with reduced suicide and self-harm in mood disorders. These benefits must be balanced against lithium’s narrow therapeutic index and its dependence on renal clearance, which creates a bidirectional relationship: kidney function shapes lithium exposure, and lithium exposure can injure the kidney. This intersection makes lithium an archetypal “shared-care” medication requiring coordinated psychiatric and nephrologic stewardship [1,2,3].

Clinical vignette: A 72-year-old woman with bipolar disorder, arterial hypertension, and type 2 diabetes mellitus had been treated with lithium for four decades. In 2018, an increase in serum creatinine to 150 μmol/L was detected; the serum lithium concentration at that time was 0.9 mmol/L, and lithium was gradually discontinued over the following two months. Nephrogenic diabetes insipidus (NDI) was also recognized during this period.

She was subsequently followed in a nephrology clinic for several years, during which serum creatinine progressively increased from 160 μmol/L (estimated glomerular filtration rate (eGFR) approximately 28 mL/min/1.73 m^2^) in 2019 to 340 μmol/L (eGFR 11 mL/min/1.73 m^2^) by late 2025. During this period, chronic kidney disease (CKD) was attributed predominantly to probable diabetic nephropathy—supported by inactive urinary sediment and estimated daily proteinuria of 1.50 g/day/1.73 m^2^—without full recognition of the contribution of chronic lithium nephropathy.

In December 2025, she was acutely admitted with hemodynamic compromise, with a blood pressure of 65/40 mmHg, and acute-on-chronic kidney injury, with serum creatinine 570 μmol/L and eGFR 6 mL/min/1.73 m^2^, accompanied by reduced responsiveness. In retrospect, the precipitating mechanism was predictable. She had become increasingly sedated under her psychotropic regimen—particularly quetiapine and lorazepam, both of which may have amplified central nervous system depressant effects in elderly patients with uremia and volume depletion. This led to reduced alertness and minimal fluid intake despite her NDI-driven obligatory need to excrete large urine volumes. The net result was a polypharmacy-related sedation syndrome that suppressed thirst and reduced voluntary fluid intake to almost zero. Consequently, she had no remaining physiological reserve against volume depletion.

The situation was further aggravated by ongoing antihypertensive therapy that included both a renin–angiotensin-system blocker and a thiazide diuretic—two drug classes that may exacerbate hypovolemia and hemodynamic renal vulnerability. Renal ultrasonography confirmed atrophic kidneys with increased cortical echogenicity and parenchymal cysts—the latter a recognized feature of chronic lithium nephropathy rather than diabetic nephropathy. Lithium had been discontinued eight years earlier; nevertheless, CKD continued to progress, and by early 2026 she was being prepared for initiation of hemodialysis.

This case did not arise from a failure to refer the patient to nephrology; nephrology was involved throughout. Rather, it arose from a gap in integrated management: NDI was recognized, but was not acted upon as a signal to intensify shared decision-making; the attribution of CKD to diabetes and hypertension was not re-evaluated, despite concurrent progression of lithium-associated nephropathy; potentially interacting medications were continued; the risk of sedation-related dehydration in a patient with obligate polyuria and a high cumulative burden of sedatives or central nervous system depressants was not explicitly anticipated; and there was no structured framework for weighing the renal trajectory against psychiatric risk. Despite lithium discontinuation eight years earlier, renal impairment continued to progress. It is precisely to address this gap that the present review proposes an integrated psychiatric–nephrologic shared-care pathway.

## 2. Historical Milestones and Contemporary Indications

### 2.1. From Cade (1949) [4] to Contemporary Psychiatric Practice

Lithium is administered clinically as lithium salts, which deliver the pharmacologically active Li^+^ ion. In this form, lithium represents one of the oldest still-used pharmacological treatments in modern psychiatry. Its contemporary psychiatric use traces back to John Cade’s seminal 1949 report describing clinical improvement in patients with “psychotic excitement”, an observation that led to the introduction of lithium for acute mania and subsequently to its use in long-term prophylaxis [4]. Over the following decades, lithium became established as a core mood stabilizer in bipolar disorder. In selected lithium-responsive patients, long-term maintenance treatment can support sustained remission, relapse prevention, and preservation of psychosocial functioning across much of adult life. This enduring clinical role is further supported by real-world observational evidence showing that lithium maintenance therapy remains highly effective compared with other mood stabilizers, particularly when used as monotherapy in routine clinical practice [5].

### 2.2. Mechanisms of Action in the Brain: Multi-Target Intracellular Pharmacology

Lithium’s therapeutic effects are best understood as the result of convergent intracellular actions rather than binding to a single receptor target. A central pharmacodynamic framework involves modulation of glycogen synthase kinase-3 (GSK-3) and inhibition of inositol monophosphatase within the phosphatidylinositol/inositol signaling pathway. Through interference with these magnesium-dependent enzymatic processes, lithium influences downstream signaling networks involved in synaptic plasticity, neurotrophic signaling, neuronal survival, apoptosis, stress-response regulation, and circadian biology [6].

These pleiotropic intracellular effects provide a biologically plausible basis for lithium’s mood-stabilizing properties in bipolar disorder. Clinically, they are relevant within a narrow therapeutic window, where maintenance efficacy must be balanced against tolerability and long-term safety.

## 3. Renal Handling of Lithium

### 3.1. Why Exposure Is Kidney-Dependent

Lithium is freely filtered at the glomerulus and undergoes tubular reabsorption in parallel with sodium, making lithium concentrations highly sensitive to changes in volume status and sodium handling. Approximately 60–80% of filtered lithium is reabsorbed in the proximal tubule via sodium channels, with a smaller fraction reabsorbed in the loop of Henle and the collecting duct. Because the proximal tubule cannot reliably distinguish lithium from sodium, any physiological or pharmacological state that promotes avid sodium reabsorption will simultaneously increase lithium reabsorption and reduce its urinary excretion [7,8].

This mechanism explains why dehydration—whether from inadequate fluid intake, vomiting, diarrhea, or febrile illness with increased insensible losses—consistently ranks among the most common precipitants of lithium toxicity in clinical practice. When extracellular volume contracts, the kidney responds by upregulating proximal tubular sodium reabsorption, lithium follows, plasma levels rise, and the therapeutic window is breached. The elderly are particularly vulnerable, as age-related declines in glomerular filtration rate (GFR), reduced thirst sensation, and diminished renal reserve mean that even modest volume depletion can produce clinically significant lithium accumulation [9].

Several drug classes exploit the same pathway and deserve specific mention. Thiazide diuretics act on the distal convoluted tubule and cause compensatory upregulation of proximal sodium and thus lithium reabsorption, reliably increasing steady-state lithium levels by 30–50%. Non-steroidal anti-inflammatory drugs (NSAIDs) reduce renal prostaglandin synthesis, leading to afferent arteriolar vasoconstriction, a fall in GFR, and decreased lithium clearance. Even short-term NSAID use for intercurrent pain or fever in a lithium-treated patient carries meaningful risk. Angiotensin-converting enzyme inhibitors and angiotensin receptor blockers attenuate the renin–angiotensin–aldosterone axis, reduce efferent arteriolar tone, and lower GFR while simultaneously promoting proximal tubular sodium retention, creating a dual mechanism for lithium accumulation. In contrast, loop diuretics have a comparatively modest effect on lithium levels because they act distally to the primary site of lithium reabsorption, though they are not without risk in the context of volume depletion [7,8,10].

It is also worth noting that long-term lithium use itself impairs renal concentrating ability—primarily through NDI caused by downregulation of aquaporin-2 channels in the collecting duct. The resulting polyuria and compensatory polydipsia create a state of chronic vulnerability whereby any interruption in fluid intake, such as during acute illness, can rapidly shift the balance toward toxicity. Monitoring of renal function, urinary osmolality, and serum lithium levels is therefore not a bureaucratic formality but a clinical necessity grounded in the drug’s renal pharmacology [11,12].

### 3.2. Target Serum Concentrations: Efficacy vs. Safety

Current recommendations from the International Society for Bipolar Disorders/International Group for the Study of Lithium-treated Patients support a standard maintenance serum lithium concentration of 0.60–0.80 mmol/L in adults, with individualized adjustment according to clinical response, tolerability, age, and medical comorbidity (lower in those with good response but poor tolerability; higher in those with insufficient response and good tolerance) [3]. In older patients and those with established CKD, a lower target—around 0.40–0.60 mmol/L—is often appropriate, balancing maintained efficacy against the narrowing margin between therapeutic and toxic concentrations as renal clearance declines. This guidance is clinically relevant for kidney safety because higher exposure and intoxication episodes are linked to adverse renal outcomes [11,12]. Once-daily dosing regimens, which prolong the trough interval between peaks despite higher Cmax, are associated with lower rates of tubular toxicity compared with divided dosing and should be considered as a renoprotective strategy in long-term users [7].

### 3.3. Mechanisms of Lithium Renal Injury: Collecting-Duct Biology as a Therapeutic Target

Experimental data support epithelial sodium channel (ENaC) as a key portal for lithium entry into collecting duct principal cells. In a transgenic mouse model lacking αENaC in the collecting duct, lithium exposure produced markedly attenuated polyuria compared with controls, supporting a causal role for ENaC-mediated entry in lithium-induced NDI [13].

ENaC is a heterotrimeric channel composed of α, β, and γ subunits, expressed on the apical membrane of principal cells in the late distal tubule and collecting duct, where its primary physiological role is aldosterone-regulated sodium reabsorption. Lithium, as a monovalent cation with an ionic radius similar to that of sodium, exploits this channel for cellular entry. Once inside the principal cell, lithium inhibits glycogen synthase kinase-3β (GSK-3β) and interferes with several downstream signaling steps that are critical for aquaporin-2 (AQP2) trafficking to the apical membrane. Notably, ENaC-mediated entry is relatively slow compared with renal tubular sodium handling in general, which may partly explain why lithium toxicity at the collecting duct is cumulative and dose/duration-dependent rather than acute. The selectivity of this entry mechanism also explains why loop diuretics, which act at the thick ascending limb, do not substantially alter lithium transport, whereas amiloride—acting directly at ENaC on the apical membrane of principal cells—reduces lithium entry at its source [13].

### 3.4. Aquaporin-2 Downregulation and Vasopressin Resistance

Lithium reduces aquaporin-2 (AQP2) expression in the renal medulla and disrupts vasopressin-regulated water transport, providing a mechanistic basis for impaired urinary concentration and NDI. Seminal experimental work by Marples et al. demonstrated marked downregulation of AQP2 protein in rat kidney medulla after chronic lithium exposure, paralleling the degree of polyuria and the reduction in urinary concentrating capacity [14]. Subsequent studies by Kortenoeven et al. showed that lithium reduces *AQP2* transcription through a mechanism that is independent of prostaglandins, pointing instead to direct intracellular signaling interference [15].

To understand this mechanism, it is necessary to briefly review normal vasopressin signaling. Vasopressin (antidiuretic hormone, ADH) binds to V2 receptors on the basolateral membrane of collecting duct principal cells, activating adenylyl cyclase, raising intracellular cyclic AMP (cAMP), and activating protein kinase A (PKA). PKA phosphorylates AQP2 at Ser256, triggering translocation of AQP2-bearing vesicles from the cytoplasm to the apical membrane, where AQP2 forms tetrameric water channels and enables osmotic water reabsorption into the hypertonic medullary interstitium. Lithium disrupts this cascade at multiple levels. First, intracellular lithium inhibits GSK-3β and other kinases involved in AQP2 phosphorylation and vesicle trafficking. Second, lithium reduces cAMP accumulation in collecting duct cells, blunting the PKA signal. Third, there is evidence that lithium downregulates *AQP2* gene transcription itself, via effects on transcription factor binding to the *AQP2* promoter, in a manner not rescued by exogenous vasopressin or prostaglandin inhibition [14,15]. The consequence is a state of acquired vasopressin resistance: circulating ADH levels may be elevated (the hypothalamic response to hypertonicity is intact), but the collecting duct cannot mount the normal concentrating response because AQP2 expression and apical trafficking are both impaired. This pattern distinguishes lithium-induced NDI from central (hypothalamic) diabetes insipidus and is diagnostically relevant: a water-deprivation test followed by exogenous desmopressin (DDAVP) will fail to elicit a rise in urine osmolality in NDI, whereas central diabetes insipidus responds to DDAVP.

### 3.5. Why Amiloride Fits the Mechanism

Because ENaC is implicated in lithium’s principal-cell entry, amiloride—a potassium-sparing diuretic that directly blocks ENaC at the apical membrane—represents a mechanistically targeted intervention for lithium-related polyuria/NDI. By reducing lithium influx into principal cells, amiloride limits intracellular lithium accumulation and thereby attenuates downstream disruption of the vasopressin–AQP2 axis. Bedford et al. demonstrated in a clinical study that amiloride improved urinary concentrating parameters in lithium-treated patients with NDI, with an increase in maximum urine osmolality after water deprivation and DDAVP challenge, consistent with partial restoration of AQP2-mediated water transport [16].

An important corollary of this mechanism is that amiloride may not only relieve symptoms of polyuria but could theoretically reduce the cumulative intracellular lithium burden in principal cells over time, potentially offering some protection against progressive collecting-duct injury. This hypothesis has not been formally tested in long-term clinical trials, and mechanistic plausibility should not be conflated with proven nephroprotection. Nonetheless, it provides a conceptual rationale for considering amiloride early in the course of lithium-related polyuria, rather than reserving it solely for severe or refractory NDI. Amiloride has been shown to attenuate lithium-induced AQP2 transcriptional downregulation independently of its ENaC-blocking action, which may partly explain why the concentrating response is improved but often not fully normalized on treatment.

## 4. Spectrum of Renal Effects: Phenotypes, Mechanisms, and Consequences

Four clinically distinct renal phenotypes are recognized in patients receiving long-term lithium therapy: (1) polyuria and nephrogenic diabetes insipidus (NDI), (2) acute lithium toxicity with acute kidney injury, (3) chronic tubulointerstitial nephropathy with progressive CKD, and (4) renal microcysts on magnetic resonance imaging (MRI). Clinical features, key mechanisms, consequences, and high-yield actions for each phenotype are summarized in Table 1.

### 4.1. Polyuria and Nephrogenic Diabetes Insipidus

The most frequent renal adverse effect of lithium is impaired urinary concentrating ability, presenting clinically as polyuria and polydipsia and, in a subset of patients, as frank nephrogenic diabetes insipidus (NDI). Prevalence estimates across cohorts range from approximately 20% to 70% depending on case definition, duration of therapy, and whether formal water-deprivation testing is performed. Clinically significant polyuria (urine output > 3 L/day) is encountered in roughly 40% of long-term users [9,10]. NDI arises because lithium enters collecting duct principal cells via the epithelial sodium channel (ENaC) and disrupts the vasopressin–aquaporin-2 (AQP2) signaling axis, preventing osmotic water reabsorption in the medullary collecting duct despite adequate circulating antidiuretic hormone (this mechanism is elaborated in Section 3). The concentrating defect is typically dose- and duration-dependent and may persist for months or years after lithium discontinuation, particularly in patients with longer treatment histories [9].

Clinically, the polyuria/NDI phenotype creates a cascade of downstream vulnerability. High free-water losses predispose patients to episodic dehydration and hypernatremia, particularly during febrile illness, heat exposure, or perioperative fasting, all of which simultaneously reduce renal lithium clearance and raise serum lithium concentrations. This pharmacokinetic consequence of volume depletion links the tubular concentrating defect directly to the risk of acute lithium intoxication and acute kidney injury [10]. Patients should therefore receive explicit counselling on sick-day hydration rules and the importance of temporarily increasing fluid intake during intercurrent illness [7]. Therapeutic management, including the role of amiloride, is addressed in Section 6.1.

### 4.2. Acute Toxicity and Acute Kidney Injury

Acute lithium toxicity is a medical emergency and a clinically important contributor to long-term renal injury. Lithium has a narrow therapeutic index (target trough concentrations 0.60–0.80 mmol/L for maintenance), and symptoms of intoxication—including coarse tremor, ataxia, confusion, and, in severe cases, seizures and hemodynamic instability—typically appear at concentrations above 1.5 mmol/L, though chronically exposed patients may show toxicity within or just above the therapeutic range [7]. Acute kidney injury complicates severe lithium intoxication through multiple mechanisms: direct tubular toxicity, hemodynamic compromise (volume depletion or cardiovascular instability), and secondary myoglobinuria from prolonged convulsions. A history of intoxication episodes is one of the strongest independent predictors of subsequent CKD in lithium-treated patients [11,12].

Most episodes of lithium intoxication are iatrogenic and preventable. The commonest precipitants are volume depletion (from gastrointestinal illness, fever, heat, or diuretics), addition of drugs that reduce renal lithium clearance—classically thiazide diuretics, NSAIDs, angiotensin-converting enzyme inhibitors, and angiotensin receptor blockers—and dose escalation without timely serum level reassessment [7,8,11]. Acute management, including indications for extracorporeal removal according to the Extracorporeal Treatments in Poisoning (EXTRIP) recommendations and post-dialysis rebound monitoring, is detailed in Section 6.3.

### 4.3. Chronic Tubulointerstitial Nephropathy and Chronic Kidney Disease

Long-term lithium therapy is associated with chronic tubulointerstitial nephropathy characterized histologically by tubular atrophy, interstitial fibrosis, global or focal glomerulosclerosis, and, in a minority of cases, focal segmental sclerotic changes [17]. Renal biopsy data, largely from patients referred to nephrology for declining kidney function, describe these features alongside distal tubular microcysts that are considered characteristic of chronic lithium nephropathy [17,18]. Importantly, biopsy cohorts are subject to selection bias: they overrepresent patients with significant dysfunction, and the prevalence of histological injury among all lithium-treated individuals—particularly those maintained at lower levels and without intoxication episodes—is not well established. Moreover, histological confirmation is not part of the routine diagnostic pathway for lithium-associated nephropathy; biopsy is reserved for situations in which the clinical picture is atypical or an alternative or superimposed cause of chronic kidney disease—such as suspected glomerulonephritis, diabetic nephropathy, or hypertensive nephropathy—must be excluded, rather than to establish lithium nephrotoxicity as such.

Classic longitudinal cohorts from Scandinavian specialist centres, where long-term lithium use has been systematically followed since the 1970s, document progressive decline in estimated glomerular filtration rate (eGFR) over decades, with a minority of patients—broadly 1–3% in treated populations—eventually reaching end-stage renal disease (ESRD) [17,19]. In Presne et al.’s French nephrology cohort, rate of eGFR decline averaged approximately 2–4 mL/min/1.73 m^2^ per year after nephrology referral, with treatment duration and degree of impairment at referral as the strongest prognostic variables [17]. Population-based studies provide a broader but more heterogeneous picture. The Lancet Psychiatry cohort by Clos et al. demonstrated a sustained, gradual decline in eGFR in lithium-treated patients compared with the general population over more than a decade of follow-up [20]. A 2025 JAMA Network Open study using Hong Kong territory-wide data found a statistically significant increased risk of CKD stage 3 or higher with lithium compared with valproate (adjusted hazard ratio approximately 1.4–1.5), particularly in older patients, while ESRD risk remained rare in absolute terms [21]. Conversely, other analyses suggest that a substantial part of the eGFR decline attributed to lithium in observational datasets may reflect pre-existing or concurrent CKD risk factors (hypertension, diabetes, ageing, and baseline renal vulnerability) rather than lithium exposure per se pointing to the importance of careful confounding control and individual trajectory assessment rather than population-level averages when counselling individual patients [12,20,21,22].

A clinically important corollary is that lithium discontinuation does not reliably halt or reverse CKD once established. Observational data suggest that eGFR stabilizes or improves in some patients after lithium is stopped, particularly when discontinued early in the course of renal impairment; however, in others (especially those with advanced fibrosis, a long treatment history, or prior intoxication episodes), renal function continues to decline despite cessation, as illustrated by the clinical vignette above. This trajectory is consistent with the histological picture of established interstitial fibrosis, which is largely irreversible regardless of the precipitating cause. Discontinuation therefore removes an ongoing injurious stimulus but cannot undo structural damage already sustained. This has direct implications for shared decision-making: the expectation that stopping lithium will protect or recover kidney function should be tempered, particularly in patients with CKD stage 3b or worse; however, the decision must be weighed against the psychiatric risk of treatment change rather than framed as a straightforward renoprotective intervention [11,17,19].

### 4.4. Imaging Phenotype: Renal Microcysts in Chronic Lithium Nephropathy

Renal magnetic resonance imaging (MRI) has an established role in the diagnostic work-up of patients with suspected chronic lithium nephropathy, particularly when the etiology of CKD is clinically uncertain. The characteristic finding is bilateral renal microcysts, typically 1–2 mm in diameter, located at the corticomedullary junction and within the medulla, corresponding histologically to cystically dilated distal tubules and collecting ducts. In the seminal series by Farres et al. published in Radiology, MRI detected microcysts in all patients with lithium-associated renal insufficiency who underwent imaging [18]. T2-weighted sequences are most sensitive for cyst detection, and the finding of numerous bilateral corticomedullary microcysts in a patient with a lithium exposure history provides strong supportive evidence for attributing CKD to chronic lithium nephropathy rather than to alternative diagnoses such as diabetic nephropathy, ischemic nephropathy, or IgA nephropathy.

In clinical practice, renal MRI is most useful in three scenarios: (1) when renal biopsy is contraindicated or not technically feasible and attribution of CKD to lithium would influence a shared decision about continuing or discontinuing therapy; (2) when CKD is identified in a lithium-treated patient with multiple competing etiologies and determination of the dominant cause has therapeutic implications; and (3) when the nephrology referral letter lacks a clear diagnosis and an objective finding supporting lithium nephropathy is needed to guide management. It is important to emphasize, however, that microcysts can persist after lithium cessation and their presence neither stages disease severity nor predicts rate of progression. These functions require serial eGFR and albuminuria monitoring over time. Moreover, not all patients with lithium-associated CKD have detectable microcysts on standard imaging, particularly earlier in the disease course or when cysts are small enough to fall below MRI resolution. Imaging findings should therefore be interpreted in clinical context and as a complement to longitudinal functional assessment, not a substitute for it [18].

## 5. Risk Factors and Modifiable Exposures

Renal risk is shaped by both patient factors (age, baseline kidney function, hypertension, diabetes) and treatment factors (duration, higher levels, episodes of intoxication, interacting drugs). Large observational datasets link higher lithium levels to acute kidney injury risk and, in some cohorts, increased risk of CKD stage 3 or higher compared with other mood stabilizers, while not consistently showing higher risk of advanced CKD in all settings [12,21,23]. Duration-risk relationships have also been reported in classic and modern cohorts [17,24].

## 6. Clinical Decision Points

### 6.1. Polyuria/Nephrogenic Diabetes Insipidus Pathway

When patients report polyuria/polydipsia, the first step is phenotyping and excluding common mimics (osmotic diuresis, hypercalcemia, uncontrolled diabetes). Formal evaluation should include measurement of urine osmolality after water deprivation followed by exogenous desmopressin (DDAVP): failure to achieve an adequate rise in urine osmolality after DDAVP confirms nephrogenic rather than central diabetes insipidus, consistent with lithium-related collecting-duct dysfunction. Management then focuses on minimizing lithium peaks and excess exposure, correcting triggers for dehydration, and using targeted therapy such as amiloride when symptoms are clinically significant and lithium continuation is desired [16]. Thiazides and NSAIDs can reduce urine output but can also increase lithium levels and toxicity risk; if used, lithium levels require close adjustment and monitoring [7,8]. Patients with NDI must receive explicit written instructions on sick-day fluid management, as any intercurrent illness or reduction in oral intake can rapidly precipitate volume depletion, raise serum lithium concentrations, and trigger acute toxicity.

### 6.2. Chronic Tubulointerstitial Nephropathy/Chronic Kidney Disease Pathway

No single eGFR threshold universally mandates discontinuation. Instead, decisions should integrate: (1) psychiatric benefit and relapse/suicide risk; (2) renal trajectory (rate of decline, albuminuria, toxicity history); (3) modifiable risk factors; and (4) availability and adequacy of alternative mood-stabilizing strategies. Decision analysis work has highlighted that lithium may remain preferred early in treatment despite long-term renal risks, emphasizing individualized trade-offs [25]. To operationalize this integration for day-to-day practice, Table 2 proposes threshold-based triggers for transitioning between routine monitoring, nephrology co-management, and shared decision-making, in line with the Kidney Disease: Improving Global Outcomes (KDIGO) 2024 staging criteria [26] and reported rates of lithium-associated eGFR decline [17,24].

When CKD is identified or progressing, several modifiable steps should be taken regardless of whether lithium is continued or tapered. Interacting drugs that reduce lithium clearance—particularly thiazide diuretics, NSAIDs, and renin–angiotensin system blockers—should be reviewed and, where clinically feasible, substituted or withdrawn. Target lithium levels should be reassessed and maintained at the lower end of the therapeutic range. General CKD care should follow guideline frameworks, including optimization of blood pressure, management of albuminuria, and avoidance of further nephrotoxic exposures [26]. Amiloride should be considered in patients with symptomatic polyuria/NDI, both to reduce fluid losses and to limit ongoing lithium entry into collecting duct cells. Lithium levels require more frequent monitoring as eGFR falls, since reduced renal clearance narrows the margin between therapeutic and toxic concentrations.

If the decision is made to discontinue lithium, this should be implemented gradually: abrupt cessation carries a substantially higher early recurrence risk than slow tapering, and a robust psychiatric follow-up and relapse-prevention plan must be in place before and during the taper [27]. As discussed in Section 4.3, renal surveillance should continue after discontinuation, and the expectation of functional recovery should be tempered in patients with established fibrosis.

### 6.3. Acute Lithium Toxicity Pathway: Urgent Assessment and Extracorporeal Treatment

When acute lithium toxicity is suspected, the immediate priorities are to stop lithium and any drugs that reduce its renal clearance (thiazides, NSAIDs, angiotensin-converting enzyme inhibitors and angiotensin receptor blockers), measure serum lithium concentration urgently alongside electrolytes and creatinine, and correct volume depletion with isotonic saline. Serum lithium must be measured even when the clinical picture appears to be dominated by another cause (such as reduced consciousness or hemodynamic compromise), because toxicity and the underlying precipitant frequently coexist and the lithium level directly guides decisions about extracorporeal removal [28].

Guidance on extracorporeal removal has been formalized by the EXTRIP (Extracorporeal Treatments In Poisoning) workgroup, whose 2015 systematic review and recommendations provide the current evidence base [28]. Hemodialysis is recommended when any of the following criteria are met: (1) a serum lithium concentration above 4.0 mmol/L in case of impaired kidney function; (2) a serum lithium concentration above 5.0 mmol/L, significant confusion, or an expected time to reduce the concentration below 1.0 mmol/L exceeding 36 h; (3) life-threatening neurological features (coma, seizures, respiratory failure) regardless of lithium level. Intermittent hemodialysis is the preferred modality given its superior lithium clearance. Continuous renal replacement therapy is an alternative when hemodialysis is not available or hemodynamic instability precludes it, though clearance is slower. A critical practical point is that lithium distributes extensively into intracellular and tissue compartments, and serum concentrations typically rebound after dialysis as lithium redistributes back into the plasma from these depots. Serum lithium must therefore be remeasured over 12 h after each dialysis session, and further sessions undertaken if the concentration rises above the threshold for intervention or if neurological features persist. Failure to check for rebound is a recognized source of under-treatment in clinical practice.

**Figure 1:**
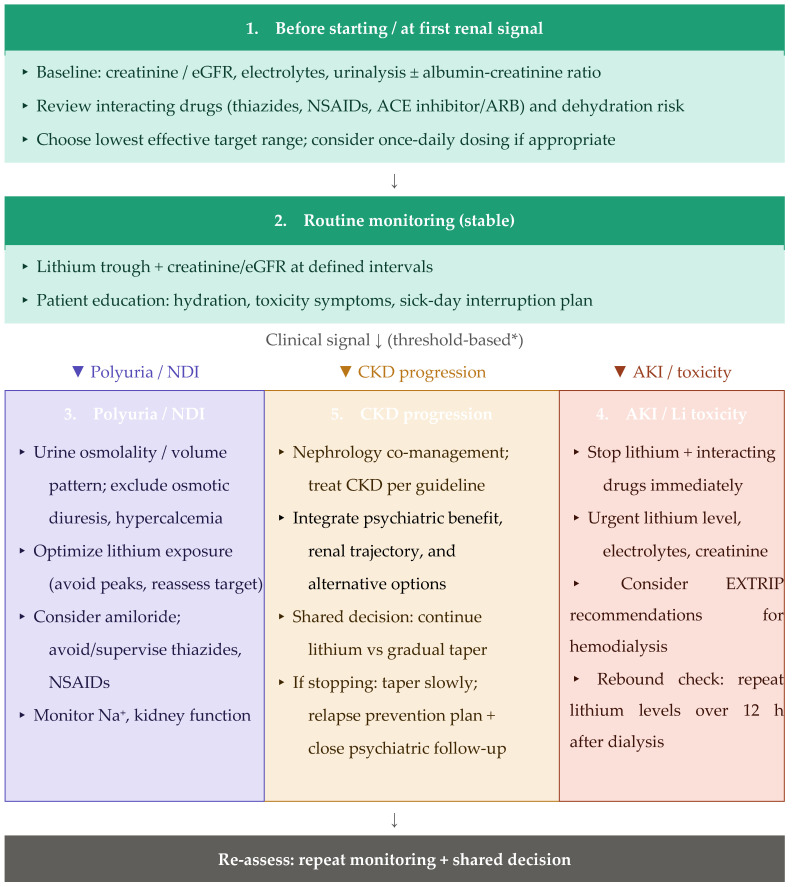
Shared-care algorithm for bipolar disorder patients on lithium with renal concerns. The algorithm is organized into five decision points: baseline and pre-treatment assessment, routine stable monitoring, management of polyuria/nephrogenic diabetes insipidus, acute toxicity and acute kidney injury, and sustained CKD progression with shared decision-making on continuation or discontinuation. All pathways require joint psychiatric–nephrologic stewardship. ACE, angiotensin-converting enzyme; AKI, acute kidney injury; ARB, angiotensin receptor blocker; CKD, chronic kidney disease; eGFR, estimated glomerular filtration rate; EXTRIP, Extracorporeal Treatments In Poisoning workgroup; NDI, nephrogenic diabetes insipidus; NSAIDs, non-steroidal anti-inflammatory drugs. * Threshold values corresponding to each transition in the pathway (routine monitoring → nephrology co-management → shared decision on continuation/taper) are detailed in Table 2.

Once the acute episode has resolved and lithium has been temporarily or permanently discontinued, the episode itself should be formally reviewed as a risk event: a toxicity episode is one of the strongest independent predictors of subsequent CKD progression, and its occurrence should prompt reassessment of the long-term benefit-risk balance, review of all precipitating factors, and—where lithium resumption is considered—explicit re-discussion of the shared-care framework and sick-day plan with both the patient and the psychiatric team (Figure 1).

## 7. A Shared-Care Model for Joint Psychiatric-Nephrology Care

### 7.1. Integrated Algorithm for Joint Psychiatric-Nephrology Care

A shared-care model for joint psychiatric-nephrology care should explicitly assign responsibilities for lithium level checks, renal surveillance, and medication reconciliation. Although exact schedules vary by jurisdiction, consensus principles include: (1) baseline kidney assessment, (2) regular lithium trough monitoring, (3) intensified monitoring after dose changes, intercurrent illness, or interacting drugs, and (4) patient education on hydration and “sick-day” interruption [7,8]. When CKD is present or emerging, general CKD care should follow international guideline frameworks (risk stratification, albuminuria assessment, mitigation of progression risk) [26]. Suggested shared-care monitoring plan is presented in Table 3.

The below presented integrated algorithm (Figure 1) organizes joint psychiatric–nephrologic care into five sequential decision points, each addressed in detail in the preceding sections. In brief, the pathway moves from baseline assessment and routine stable monitoring, through targeted management of polyuria/NDI and acute toxicity (including EXTRIP-guided decisions on extracorporeal removal), to the structured shared decision on lithium continuation versus gradual taper when CKD progresses. The algorithm is intended as a practical framework to be adapted to local policy and individual patient circumstances; its underlying evidence and clinical reasoning are elaborated in previous sections.

### 7.2. Psychopharmacological Alternatives After Lithium Discontinuation in CKD

Lithium is used in psychiatry for several clinically overlapping indications. Its clinical use includes acute mania and mixed episodes, maintenance treatment of bipolar disorder, selected strategies for bipolar depression, reduction in suicide risk in mood disorders, and augmentation in treatment-resistant unipolar depression [1,2,29,30,31,32,33,34,35]. In patients with chronic kidney disease (CKD), lithium discontinuation should therefore be considered a planned therapeutic transition rather than a simple drug withdrawal. From a psychiatric perspective, the main risk is relapse. Relapse is particularly relevant in bipolar I disorder, mixed presentations, rapid cycling, psychotic mood episodes, high previous morbidity, and suicidality. From a nephrological perspective, the decision should include CKD stage, eGFR trajectory, albuminuria, recurrent acute kidney injury, NDI, dehydration risk, frailty, comorbidities, and interacting medication [26,29].

The pace of lithium discontinuation is clinically important. The exception is urgent lithium discontinuation because of intoxication, acute kidney injury, severe dehydration, delirium, or rapidly progressive renal failure. In all other situations, gradual dose reduction and cross-titration to an alternative strategy are preferred. Recurrence after lithium discontinuation occurs early in a substantial proportion of patients. Manic recurrence often occurs earlier than depressive recurrence. Rapid discontinuation is associated with a shorter time to recurrence than gradual discontinuation [27,29,32,36,37,38]. In practice, lithium should usually be reduced over at least 4 weeks, and preferably over up to 3 months in patients with long-term exposure, bipolar I disorder, mixed features, previous severe episodes, or high suicide risk. Close monitoring is required during dose reduction and for at least 3 months after discontinuation [27,29,32,36,37,38].

Replacement pharmacotherapy should be selected according to the indication for which lithium was used and according to the predominant polarity of illness. In mania-predominant or mixed presentations, valproate/divalproex or an antimanic atypical antipsychotic such as quetiapine, aripiprazole, olanzapine, asenapine, risperidone, or cariprazine may be considered [1,29,30,31,32,33]. In bipolar illness with predominant depressive polarity, lamotrigine, quetiapine, lurasidone, or cariprazine are more relevant options [1,29,30,31,32]. Antidepressant monotherapy should be avoided in bipolar I disorder, acute manic episodes, and mixed presentations [31,32,33]. When lithium has been used as augmentation in treatment-resistant unipolar depression, the replacement strategy should follow evidence-based depression treatment pathways. These include antidepressant optimization or switching, augmentation with an atypical antipsychotic, structured psychotherapy, neuromodulation, or specialist treatment-resistant depression care [29,34,35]. In patients in whom lithium was used mainly because of high suicide risk, there is no direct pharmacological substitute for lithium’s anti-suicidal evidence. In this indication, discontinuation therefore requires explicit reassessment of suicide risk and a strengthened safety plan [2,29].

Drug choice should also account for renal pharmacokinetics and general medical vulnerability in older or frail CKD patients; dosing considerations, renal cautions, and supporting references for each agent are summarized in Table 4.

Beyond drug selection, safe lithium discontinuation requires reciprocal psychiatric-nephrological competence. Psychiatrists should recognize renal deterioration, NDI, dehydration, electrolyte disturbances, acute kidney injury, delirium, and lithium toxicity. Nephrologists should recognize that abrupt lithium withdrawal may precipitate mania, mixed affective relapse, depressive relapse, suicidality, or the need for urgent psychiatric admission. Joint education, shared monitoring protocols, and rapid communication pathways are therefore patient-safety measures, not merely optional organizational improvements [26,29,32].

## 8. Conclusions

Lithium’s clinical value in bipolar disorder remains substantial, including relapse prevention and reduced suicide risk, but its reliance on renal clearance and its potential for tubular dysfunction and chronic tubulointerstitial injury make kidney outcomes a core part of long-term treatment planning. A practical shared-care model should prioritize prevention of intoxication, early recognition and targeted management of polyuria/NDI (including ENaC-directed therapy), and CKD risk stratification with nephrology co-management when kidney function declines. When CKD emerges, discontinuation decisions should be deliberate and individualized, balancing psychiatric severity and suicide risk against renal trajectory, and—if discontinuation is chosen—implemented gradually with a robust relapse-prevention plan.

## Figures and Tables

**Table 1:** Renal phenotypes of lithium exposure (clinical framing). Abbreviations: NDI, nephrogenic diabetes insipidus; ENaC, epithelial sodium channel; AKI, acute kidney injury; CKD, chronic kidney disease; GI, gastrointestinal; EXTRIP, Extracorporeal Treatments in Poisoning; MRI, magnetic resonance imaging.

Phenotype	Typical Clinical Features	Key Mechanism(s)	Consequences	Suggested Actions
Polyuria/impaired concentration ± NDI	Polyuria, polydipsia, low urine osmolality	ENaC-mediated entry; aquaporin-2 downregulation	Dehydration, hypernatremia risk, AKI susceptibility	Consider amiloride; review dose/levels; counsel sick-day rules
Acute lithium toxicity/AKI	GI/neurologic symptoms; rising creatinine	Reduced clearance (dehydration/drug interactions)	AKI; possible residual CKD risk	Stop offenders; urgent lithium level + renal panel; consider EXTRIP recommendations
Chronic tubulointerstitial nephropathy/CKD	Gradual decline in glomerular filtration	Chronic interstitial injury/fibrosis	CKD progression; rare end-stage disease	Shared decision: continue vs. taper; nephrology co-care; avoid toxicity
Renal microcysts (imaging)	MRI renal microcysts (1–2 mm), corticomedullary	Tubular remodeling/cystic change	Diagnostic support, not a staging tool	Use to support attribution when uncertain; focus on trajectory

**Table 2:** Threshold-based triggers for nephrology involvement in lithium-treated patients with renal changes. Abbreviations: ACE, angiotensin-converting enzyme; ARB, angiotensin receptor blocker; eGFR, estimated glomerular filtration rate; KDIGO, Kidney Disease: Improving Global Outcomes; NSAID, non-steroidal anti-inflammatory drug; UACR, urine albumin-to-creatinine ratio.

Level	Laboratory/Clinical Trigger	Responsible Party	Action
A—Intensified monitoring	Isolated creatinine rise with eGFR ≥ 60 mL/min/1.73 m^2^ (KDIGO G1–G2); no new-onset albuminuria	Psychiatrist (independently)	Repeat testing in 4–6 weeks; review interacting drugs (thiazides, NSAIDs, ACE inhibitors/ARBs); reassess lithium toward lowest effective target
B—Nephrology co-management	Confirmed eGFR 30–59 mL/min/1.73 m^2^ (G3a–G3b) on two measurements ≥ 90 days apart; or eGFR decline ≥ 25% from baseline; or rate of decline > 5 mL/min/1.73 m^2^/year [17,24]; or new albuminuria ≥ A2 (UACR ≥ 30 mg/g)	Psychiatrist refers; nephrologist co-manages	Nephrology evaluation; lithium continued pending joint review
C—Shared decision: continue vs. taper	eGFR < 30 mL/min/1.73 m^2^ (G4–G5); or progression despite correction of modifiable factors; or imaging/biopsy evidence of advanced interstitial fibrosis (Section 4.3)	Psychiatrist + nephrologist + patient	Formal shared decision-making (see text above)

**Table 3:** Suggested shared-care monitoring plan. Abbreviations: CKD, chronic kidney disease; AKI, acute kidney injury; NDI, nephrogenic diabetes insipidus; eGFR, estimated glomerular filtration rate; Na^+^, sodium; K^+^, potassium. The symbol ✓ indicates assessment at baseline.

What to Monitor	Baseline	When Stable	Intensify Monitoring When…	Typical Shared-Care Response
Serum lithium (12 h trough)	✓	every 3–6 months	dose change; dehydration/illness; interacting drug; CKD	recheck in ~5–7 days; adjust dose; reinforce sick-day rules
Creatinine/eGFR	✓	every 6 months (often)	sustained decline; CKD stage ≥ 3; AKI episode	confirm trend; evaluate contributors; consider nephrology co-management
Electrolytes (Na^+^, K^+^)	✓	every 6–12 months	polyuria/NDI; diuretics; illness	treat derangements; review regimen; consider amiloride
Urinalysis ± albumin-creatinine ratio	✓ (risk-based)	yearly (risk-based)	proteinuria/hematuria	quantify albuminuria; CKD work-up per guideline

**Table 4:** Suggested psychopharmacological alternatives after lithium discontinuation in CKD.

Clinical Situation/Drug	Preferred Use/Usual Adult Dose	Renal Considerations	CKD-Relevant Cautions/Response
Lithium tapering strategy	All non-urgent discontinuations. Reduce over ≥4 weeks; slower tapering up to 3 months is appropriate in high-risk patients.	Urgent cessation may be necessary in severe intoxication, AKI, severe dehydration, delirium, or rapidly progressive renal failure.	Monitor during tapering and for at least 3 months after discontinuation for mania, depression, mixed symptoms, insomnia, agitation, and suicidality [27,29,32,36,37,38].
Valproate/Divalproex	Acute mania; mixed states; mania-predominant maintenance. Dose: 500–1500 mg/day; titrate clinically and by serum level.	Usually no routine renal dose adjustment; free fraction may increase, so total serum levels may be misleading [39].	Monitor liver function, platelets, sedation, weight and clinical toxicity; major cautions include hepatotoxicity, thrombocytopenia, hyperammonaemia, pancreatitis and reproductive risk.
Lamotrigine	Bipolar depression; depression-predominant maintenance. Dose: 100–200 mg/day; lower with valproate.	Reduced maintenance doses may be effective in significant renal impairment [40].	Titrate slowly; monitor for rash/SJS/TEN. Not suitable as sole treatment for acute mania or marked mixed activation.
Quetiapine	Mania; bipolar depression; maintenance; mixed presentations; MDD augmentation. Dose: bipolar depression 300 mg/day; mania/maintenance 400–800 mg/day; MDD augmentation 150–300 mg/day XR.	No routine renal dose adjustment is generally required [41].	Start low and titrate cautiously in older/frail CKD patients; monitor sedation, orthostasis, falls, glucose, lipids and weight.
Aripiprazole	Acute mania; mania prevention; psychosis; MDD augmentation. Dose: mania 10–30 mg/day; MDD augmentation 2–15 mg/day.	No renal dose adjustment for GFR 15–90 mL/min [42].	Monitor activation, akathisia and impulse-control symptoms; consider CYP2D6/3A4 interactions.
Olanzapine	Severe mania; agitation; psychosis; selected maintenance. Dose: 5–20 mg/day.	No renal dose adjustment; not removed by dialysis [43].	Use when strong antimanic effect is needed; monitor metabolic profile closely, especially in diabetes, obesity, dyslipidaemia or cardiovascular risk.
Asenapine	Manic or mixed episodes. Dose: 5–10 mg sublingually twice daily.	No renal dose adjustment for GFR 15–90 mL/min [44].	Monitor tolerability, sedation, EPS, QTc risk and adherence to sublingual administration; avoid severe hepatic impairment.
Cariprazine	Mania/mixed episodes; bipolar depression; MDD augmentation. Dose: mania/mixed 3–6 mg/day; bipolar depression or MDD augmentation 1.5–3 mg/day.	No adjustment in mild-moderate renal impairment; not recommended if CrCl < 30 mL/min [45].	Avoid in severe renal impairment; monitor akathisia, insomnia, CYP3A4 interactions and delayed adverse effects due to long half-life.
Risperidone	Mania; agitation; psychotic symptoms. Dose: usually 1–6 mg/day.	In severe renal impairment, start 0.5 mg twice daily and titrate slowly [46].	Monitor EPS, hyperprolactinaemia, orthostasis, sedation and accumulation risk.
Paliperidone	Psychotic or schizoaffective presentations; less central for standard bipolar replacement. Dose depends on formulation and renal function.	Dose reduction required; oral paliperidone is not recommended when CrCl < 10 mL/min [47].	Generally less attractive in advanced CKD; monitor renal accumulation, EPS, hyperprolactinaemia and QTc risk.
Lurasidone	Bipolar depression, especially when metabolic risk is important. Dose: 20–120 mg/day with food.	In moderate/severe renal impairment, start 20 mg/day and do not exceed 80 mg/day [48].	Monitor akathisia, nausea, somnolence and CYP3A4 interactions; not an acute antimanic drug.
Brexpiprazole	Augmentation in treatment-resistant MDD; not a bipolar mood stabilizer. Dose: usually 0.5–3 mg/day.	In moderate, severe, or end-stage renal impairment, maximum recommended dose is 2 mg/day for MDD [49].	Monitor akathisia, weight gain, metabolic effects and CYP2D6/3A4 interactions.
Carbamazepine/oxcarbazepine	Less preferred antimanic alternatives. Dose: carbamazepine 400–1200 mg/day; oxcarbazepine 600–1200 mg/day.	Oxcarbazepine: if CrCl < 30 mL/min, initiate at one-half usual starting dose and titrate slowly [50,51].	Generally avoid if safer options exist; monitor hyponatraemia, interactions, hepatic and haematological toxicity [1,33,50,51].
High suicide risk after lithium withdrawal	No direct pharmacological substitute for lithium; strengthen relapse prevention, safety planning, psychotherapy, family involvement and follow-up frequency.	CKD progression may force discontinuation despite psychiatric benefit.	Reassess suicide risk and monitor closely during and after taper; avoid abrupt withdrawal unless medically necessary [2,29,36,37,38].
Shared psychiatric-nephrological pathway	All CKD patients discontinuing lithium; reciprocal education, shared monitoring and rapid communication pathways.	Monitor eGFR trajectory, albuminuria, hydration, sodium, AKI episodes, NDI and interacting drugs.	Delayed recognition of lithium toxicity, AKI, dehydration, delirium, severe relapse or suicidal crisis may become life-threatening [26,29,32].

Note: Dose ranges are usual adult psychiatric dose ranges and do not replace individualized prescribing. In advanced CKD, dialysis, older age, frailty, polypharmacy or unstable medical conditions, lower starting doses, slower titration and closer monitoring are often appropriate. Abbreviations: AKI, acute kidney injury; CKD, chronic kidney disease; CrCl, creatinine clearance; CYP, cytochrome P450; eGFR, estimated glomerular filtration rate; EPS, extrapyramidal symptoms; GFR, glomerular filtration rate; MDD, major depressive disorder; NDI, nephrogenic diabetes insipidus; QTc, corrected QT interval; SJS, Stevens-Johnson syndrome; TEN, toxic epidermal necrolysis; XR, extended release.

## Data Availability

No new data were created or analyzed in this study. Data sharing is not applicable to this article.

## Abbreviations

The following abbreviations are used in this manuscript:

NDI	Nephrogenic diabetes insipidus
CKD	Chronic kidney disease
AKI	Acute kidney injury
ENaC	Epithelial sodium channel
AQP2	Aquaporin-2
eGFR	Estimated glomerular filtration rate
ESRD	End-stage renal disease
EXTRIP	Extracorporeal Treatments in Poisoning workgroup
GFR	Glomerular filtration rate
GSK-3β	Glycogen synthase kinase-3β
MRI	Magnetic resonance imaging
NSAIDs	Non-steroidal anti-inflammatory drugs
